# Ultrasound-Enhanced Assessment of Vitreous Status in Exudative AMD: Associations with Neovascular Phenotypes, Treatment Burden, and Functional Outcomes

**DOI:** 10.3390/jcm15010167

**Published:** 2025-12-25

**Authors:** Cristina Rodriguez-Vidal, Lucía Galletero Pandelo, Nerea Martínez-Alday, Manuel Bande, María José Blanco Teijeiro

**Affiliations:** 1 Department of Ophthalmology, Hospital de la Santa Creu i Sant Pau, 08025 Barcelona, Spain; 2 Department of Medicine and Surgery, University of Santiago de Compostela, 15782 Santiago de Compostela, Spain; 3 Department of Ophthalmology, Hospital Universitario de Basurto, 48013 Bilbao, Spain; 4 Department of Ophthalmology, Hospital Universitario de Cruces, 48903 Barakaldo, Spain; 5 Department of Ophthalmology, Complejo Hospitalario Universitario de Santiago de Compostela (CHUS), 15782 Santiago de Compostela, Spain

**Keywords:** posterior vitreous detachment, age-related macular degeneration, B-scan ultrasound, vitreoretinal interface, anti-VEGF therapy

## Abstract

**Background/Objectives:** The influence of the vitreoretinal interface on neovascular age-related macular degeneration (nAMD) remains poorly characterized. Most previous studies relied solely on macular optical coherence tomography (OCT), which provides limited information about global posterior vitreous detachment (PVD). This study evaluated (1) whether ultrasonography-defined PVD status differs between nAMD eyes and healthy controls, and (2) whether baseline PVD influences macular neovascularization (MNV) phenotype and functional outcomes following anti-vascular endothelial growth factor (anti-VEGF) therapy. **Methods:** In this prospective longitudinal study, treatment-naïve nAMD eyes and population-based healthy controls underwent dynamic B-scan ultrasonography and spectral-domain OCT. PVD was categorized as absent, partial, or complete. nAMD eyes received intravitreal aflibercept according to a treat-and-extend protocol and were followed for 12 months. Structural parameters—including subretinal fluid (SRF), intraretinal fluid (IRF), and central foveal thickness—along with best-corrected visual acuity (BCVA) were recorded. A multivariable linear regression model was performed to assess whether PVD independently predicted BCVA gain after adjusting for age, baseline BCVA, MNV subtype, SRF, atrophy, and number of injections. **Results:** Absence of PVD was significantly more frequent in nAMD eyes than in controls (*p* < 0.001), whereas complete PVD prevalence was comparable. In nAMD, absence of PVD was associated with a higher prevalence of MNV type 2 (*p* = 0.032), while partial/complete PVD correlated with type 1 lesions. After 12 months, eyes without PVD achieved the greatest visual improvement (mean BCVA gain +0.34 ± 0.26), outperforming eyes with complete PVD (*p* = 0.026). A multivariable model confirmed that absence of PVD was an independent predictor of greater BCVA gain (β = −0.27; 95% CI −0.42 to −0.12; *p* = 0.0008). Eyes with complete PVD required more injections (*p* = 0.046). SRF and foveal-thickness reductions occurred across groups, whereas IRF changes were similar. **Conclusions:** Ultrasonography-defined PVD status differs markedly between nAMD and healthy eyes and independently influences neovascular phenotype and functional response to anti-VEGF therapy. These findings underscore the physiological importance of the vitreoretinal interface and support the use of ocular ultrasonography as an adjunct tool for assessing global vitreous status in selected nAMD settings.

## 1. Introduction

Age-related macular degeneration (AMD) is the leading cause of irreversible visual loss among older adults in developed countries [1]. Its exudative form, neovascular age-related macular degeneration (nAMD), characterized by the development of macular neovascularization (MNV), requires chronic anti–vascular endothelial growth factor (anti-VEGF) therapy; however, both anatomical and functional responses show substantial inter-individual variability [2,3,4,5]. This variability suggests the presence of physiological modulators that remain insufficiently defined.

Among these potential modulators, the vitreoretinal interface has received increasing attention. Altered posterior vitreous detachment (PVD) patterns—including persistent vitreous attachment (absence of complete detachment), vitreomacular adhesion (VMA), and vitreomacular traction (VMT)—have been proposed as contributors to angiogenesis, cytokine distribution, and therapeutic response [6,7,8]. However, current evidence is heterogeneous and often contradictory. A major limitation is that most studies assess PVD exclusively through macular optical coherence tomography (OCT), a technique restricted to the foveal region and poorly suited to characterize peripheral or global vitreous separation [9].

In this context, ocular ultrasonography—a classical method for evaluating the posterior vitreous—provides complete visualization of the vitreous body and global posterior vitreous detachment, offering complementary information to macular OCT, which is limited to the foveal region [10]. Despite these advantages, ultrasonography has been scarcely integrated into studies of neovascular AMD.

Understanding how the global status of posterior vitreous detachment influences neovascular activity, macular neovascularization phenotypes, and response to anti-VEGF therapy is crucial to improving clinical stratification and advancing toward more personalized therapeutic strategies. Accordingly, the present study focuses on the role of ultrasonography as a complementary tool for assessing global vitreous status in selected prognostic and research scenarios—particularly for stratifying functional trajectory and treatment burden under standardized anti-VEGF regimens—rather than as a routine clinical imaging modality.

## 2. Materials and Methods

### 2.1. Study Design

We conducted a prospective, longitudinal, single-center study evaluating the influence of PVD status on structural and functional outcomes in treatment-naïve patients with nAMD. The study focused on the relationship between the vitreoretinal interface, neovascular membrane phenotype, and response to intravitreal anti-VEGF therapy over a 12-month follow-up period.

### 2.2. Setting and Ethics Approval

The study was carried out at the Department of Ophthalmology, Hospital Universitario de Cruces (Barakaldo, Bilbao, Spain). All examinations and procedures adhered to the principles of the Declaration of Helsinki (2013). Ethical approval was granted by the Institutional Review Board (CEIm OSI Ezkerraldea–Enkarterri–Cruces; protocol code E22/11; approval date: 29 March 2022). Written informed consent was obtained from all participants prior to enrolment.

### 2.3. Participants

Consecutive patients newly diagnosed with nAMD between March 2022 and August 2023 were screened for inclusion. Eligible subjects were ≥50 years old, had a de novo diagnosis confirmed by clinical examination and OCT, and had not received previous intravitreal treatment. Exclusion criteria included history of vitreoretinal surgery, significant ocular trauma, high myopia (>6 diopters), active inflammatory or neoplastic ocular disease, or any condition limiting adequate imaging acquisition. Only the first diagnosed eye per patient was included to prevent inter-eye correlation.

### 2.4. Control Group Selection

Controls were recruited consecutively from the same outpatient clinics and met the same inclusion/exclusion criteria described above, except for the absence of macular disease. Normal macular anatomy was confirmed on spectral-domain OCT. Controls were frequency-matched to the nAMD cohort by age and sex.

### 2.5. Variables and Assessments

At baseline, all participants underwent a comprehensive ophthalmic evaluation including detailed medical history, best-corrected visual acuity (BCVA) using a Snellen chart, refraction, intraocular pressure measurement, and slit-lamp biomicroscopy. Axial length (AXL) was obtained using optical biometry (IOLMaster 700, Carl Zeiss Meditec, Jena, Germany). Systemic variables included age, sex and relevant comorbidities (arterial hypertension, diabetes mellitus, connective-tissue disorders). Ocular history (prior surgeries, high myopia >6D, previous uveitis or laser treatments) was recorded.

Macular structure was assessed using spectral-domain optical coherence tomography (Spectralis OCT, Heidelberg Engineering, Heidelberg, Germany) with high-definition LINE scans (240-µm spacing) and CUBE volumetric acquisition. OCT was used to confirm the diagnosis and to quantify foveal thickness, presence of subretinal fluid (SRF), intraretinal fluid (IRF), and pigment epithelial detachment. All scans were obtained by an experienced operator under standardized illumination and pharmacological mydriasis, and reviewed using proprietary segmentation software to ensure consistency.

OCT and ultrasonography played complementary but distinct roles in the study design. Spectral-domain OCT was used to characterize macular morphology and retinal biomarkers, whereas ocular ultrasonography was the reference method for assessing global vitreous status and defining posterior vitreous detachment categories. OCT was not used to classify posterior vitreous detachment but was employed in selected cases to determine the presence of VMA on OCT in eyes initially classified as partial PVD by ultrasonography. Ultrasonography provides a validated dynamic assessment of global PVD status, particularly for identifying complete vitreous detachment and posterior hyaloid mobility beyond the macular region, but has limited spatial resolution for defining focal macular adhesion; therefore, OCT was considered the reference technique for vitreomacular attachment.

### 2.6. Ultrasound-Based Assessment of PVD

PVD was assessed using standardized ocular ultrasonography (Quantel Compact Touch, 10-MHz probe, Quantel Medical, Clermont-Ferrand, France) including static B-scan and dynamic kinetic evaluation. After instillation of topical anesthesia, the probe was placed directly on the bulbar conjunctiva using sterile ophthalmic gel as coupling medium.

For each eye, six dynamic B-scan projections were obtained in a systematic fashion: four transverse (superior, inferior, temporal, nasal) and two longitudinal (temporal and nasal). Real-time kinetic evaluation in primary position and during small eye movements enabled assessment of posterior hyaloid mobility and detection of vitreoretinal adhesions.

Based on visibility of the posterior cortical vitreous, mobility patterns, and the presence or absence of a Weiss ring, PVD status was categorized using a predefined three-stage ultrasound system as follows:

- No PVD: no identifiable hyaloid separation; homogeneous vitreous cavity.
- Partial PVD: localized or broad separation with persistent adhesion at the macula and/or optic disc.
- Complete PVD: hyper-echoic membrane fully detached from the posterior pole in all projections, with or without a Weiss ring.

This operational system is consistent with established models of age-related PVD progression and prior descriptions of its early stages [11,12,13].

### 2.7. Clinical Management and Follow-Up

Patients were treated with intravitreal aflibercept (Eylea^®^, Bayer AG, Leverkusen, Germany) following a standardized treat-and-extend regimen. Follow-up visits were scheduled at months 1, 2, 3, 6, 9, and 12, and included BCVA reassessment, macular OCT, and repeat ultrasonography to evaluate vitreous changes over time. The total number of injections, interval extension, and anatomical response were recorded.

### 2.8. Ultrasound Image Evaluation

B-scan ultrasonography was independently graded by two masked readers (C.R.-V., M.B.). Disagreements were adjudicated by a senior specialist (M.J.B.T.). Interobserver agreement was excellent (Cohen’s κ = 0.92).

OCT images were also reviewed independently by the same graders. No discrepancies occurred in OCT-based assessments, yielding perfect interobserver concordance (κ = 1.00).

### 2.9. Unit of Analysis

The primary unit of analysis was the eye, with only one eye per patient included to maintain statistical independence.

### 2.10. Statistical Analysis

Sample size was determined pragmatically based on the expected incidence of treatment-naïve neovascular AMD during the recruitment period and on prior exploratory studies evaluating vitreous status in AMD. Given the limited existing data on ultrasonography-defined PVD as a prognostic factor, this prospective study was designed as hypothesis-generating rather than powered for a predefined effect size.

Continuous variables were expressed as mean ± standard deviation or median (interquartile range), according to their distribution. Categorical variables were summarized as frequencies and percentages. Between-group comparisons (no PVD vs. partial PVD vs. complete PVD) were performed using the Student’s *t*-test or Mann–Whitney U test for continuous variables, and Pearson’s χ^2^ or Fisher’s exact test for categorical variables, as appropriate.

Longitudinal changes in structural parameters (SRF, IRF, and central retinal thickness) and functional outcomes (BCVA) were evaluated descriptively at baseline, 3, 6, and 12 months. Given the heterogeneous evolution of anatomical variables and the non-uniform distribution of measurements, repeated-measures analyses were used only for exploratory purposes.

BCVA was measured using a Snellen chart and converted to logMAR for all analyses. For clinical interpretability, logMAR differences were additionally expressed as approximate ETDRS-letter equivalents in the Results and Discussion, where appropriate.

To determine whether vitreous status independently influenced the functional response to therapy, a multivariable linear regression model was constructed using BCVA gain at 12 months (BCVA_12_m—BCVA_baseline) as the dependent variable. Covariates were prespecified based on biological plausibility and prior literature and included: baseline PVD status (present vs. absent), age, sex, baseline BCVA, MNV subtype (type 2 vs. other), presence of SRF at baseline, baseline atrophy, and total number of intravitreal injections during follow-up. Assumptions of linearity, homoscedasticity, and normality of residuals were verified. Multicollinearity was assessed using the variance inflation factor (VIF), with all VIF values < 1.5 indicating absence of meaningful collinearity.

All statistical tests were two-sided, and a *p*-value < 0.05 was considered statistically significant. Statistical analyses were performed using R software (version 4.5.2; R Foundation for Statistical Computing, Vienna, Austria; https://www.r-project.org, accessed on 31 October 2025). Data import, management, and statistical modeling were conducted using the R packages readxl, dplyr, car, and broom.

## 3. Results

Results are presented in the following order: first, a comparison of vitreous status between nAMD eyes and healthy controls; second, the association between baseline vitreous configuration and MNV phenotype; third, the impact of baseline vitreous status on anatomical and functional outcomes; and finally, differences in treatment burden according to vitreous status.

### 3.1. Comparison with a Control Sample

A total of 51 eyes with nAMD and 65 healthy control eyes were included. Baseline demographic and ocular characteristics—including age, sex, AXL, hypertension, and prior cataract surgery—were comparable between groups, with no statistically significant differences (all *p* > 0.30) (Table 1).

The distribution of posterior vitreous detachment differed markedly between groups (*p* = 0.008). Absence of PVD was significantly more frequent in nAMD eyes (25.5%) compared with controls (6.2%). Partial PVD was more common in healthy subjects (40.0%) than in nAMD eyes (23.5%), while complete PVD showed a similar prevalence in both groups (51.0% vs. 53.8%).

These findings indicate that eyes with nAMD display a higher proportion of attached vitreous and a lower frequency of partial PVD compared with age-matched controls.

### 3.2. Descriptive Analysis of the nAMD Group

Baseline clinical and OCT characteristics of the nAMD cohort are summarized in Table 2. These baseline functional and structural parameters served as the reference for longitudinal outcome analyses.

Baseline OCT features were consistent with active neovascular disease: type 1 MNV in 54.9%, subretinal fluid (SRF) in 78.4%, intraretinal cysts in 41.2%, and retinal atrophy in 39.2% (Table 2).

### 3.3. Vitreous Detachment Status and Clinical Profile in nAMD

Baseline demographic and clinical characteristics stratified by PVD status are summarized in Table 3. No statistically significant differences were observed across PVD groups for demographic variables, baseline visual acuity, axial length, or OCT-derived structural parameters.

Baseline retinal morphology—including MNV type, intraretinal cysts, SRF, and atrophy—also showed no statistically significant differences among the three PVD groups, although variability in distribution was evident (Table 3).

Because partial and complete PVD did not differ in baseline characteristics, both categories were merged into a single group (“PVD present”) to improve statistical power. When analyzed this way, a significant association emerged between vitreous status and neovascular phenotype: type 1 MNV was more frequent in eyes with PVD (63.2%) than in those without PVD (30.8%) (*p* = 0.043) (Table 4).

To further refine this observation, OCT scans of eyes classified as partial PVD were reviewed. In 5 of 12 eyes, the posterior hyaloid remained attached to the macula. Accordingly, ultrasonography provided the global PVD classification, whereas OCT was used only to characterize VMA within the partial-PVD subgroup. Reclassifying eyes according to VMA on OCT (present vs. absent) strengthened the association between vitreous status and neovascular phenotype (*p* = 0.022), with type 1 MNV more prevalent among eyes with complete hyaloid detachment.

### 3.4. Vitreous Dynamics and Clinical Changes During Follow-Up

A progressive shift in vitreous status was observed throughout the 12-month follow-up. At baseline, roughly one quarter of patients exhibited no PVD and another quarter showed partial detachment, while slightly more than half already presented complete PVD. By month 3, the proportions of eyes without PVD or with partial PVD had begun to decline, and complete detachment had become the predominant pattern in more than two-thirds of the cohort. This trend continued at month 6, with complete PVD exceeding 70% of cases. By month 12, vitreous status had largely stabilized, with just over 10% of eyes still showing no PVD, a similar proportion presenting partial PVD, and nearly three quarters displaying complete detachment. This evolution toward complete PVD was statistically significant (*p* < 0.001).

Substantial anatomical improvement was observed during follow-up, with a marked overall reduction in exudative activity. Subretinal fluid (SRF) decreased from 78.4% at baseline to 9.8% at month 12 (*p* < 0.001), and intraretinal fluid (IRF) declined from 41.2% to 13.7% (*p* < 0.001).

Central foveal thickness decreased from 409 ± 122 µm at baseline to 272 ± 54 µm at month 6 and remained stable at month 12 (275 ± 62 µm; *p* < 0.001). In contrast, retinal atrophy increased from 39.2% to 54.9% over follow-up (*p* < 0.001).

### 3.5. Association Between Baseline Vitreous Status and the Evolution of Clinical Parameters

**Figure 1:**
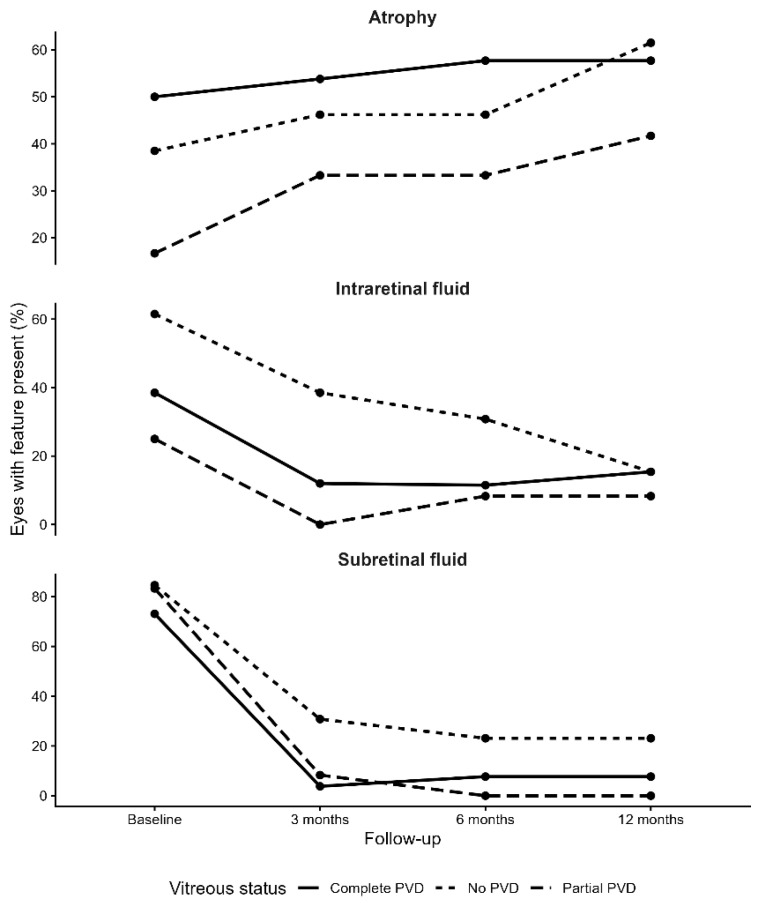
Longitudinal evolution of retinal atrophy, intraretinal fluid, and subretinal fluid according to baseline vitreous status (no posterior vitreous detachment, partial posterior vitreous detachment, and complete posterior vitreous detachment) over 12 months of follow-up. Values represent the percentage of eyes in which each feature was present at each time point.

When longitudinal changes were stratified by baseline vitreous configuration, no significant differences were observed in the magnitude of central foveal thickness reduction between groups (*p* = 0.683). Subgroup differences were driven mainly by SRF dynamics, whereas IRF improvement and atrophy progression were broadly comparable across vitreous-status categories (Figure 1). SRF-free status increased from 15.4% to 76.9% in eyes without PVD, reached 100% in the partial-PVD group, and increased from 26.9% to 92.3% in the complete-PVD group. Although improvement was numerically greatest in the partial-PVD group, the difference reached statistical significance only when comparing partial PVD with no PVD (*p* = 0.027), with no other intergroup contrasts achieving significance.

Visual outcomes showed clearer differentiation. At month 6, eyes without PVD displayed a tendency toward greater functional improvement, although the difference did not reach statistical significance. By month 12, however, the pattern became more distinct: eyes without PVD achieved the largest gain in BCVA, significantly outperforming those with complete PVD (*p* = 0.026). When vitreous status was reassessed using macular OCT in eyes initially classified as partial PVD, the contrast became even more marked. Eyes with persistent VMA on OCT achieved substantially better visual outcomes than those with complete hyaloid detachment (+0.29 ± 0.21 vs. +0.04 ± 0.18; *p* < 0.001), suggesting that vitreous attachment may exert a favorable mechanical or biological influence on visual recovery.

No significant correlation was found between BCVA gain and the degree of retinal thinning (r = −0.098; *p* = 0.500), indicating that functional improvement was not simply a reflection of changes in foveal thickness.

**Figure 2:**
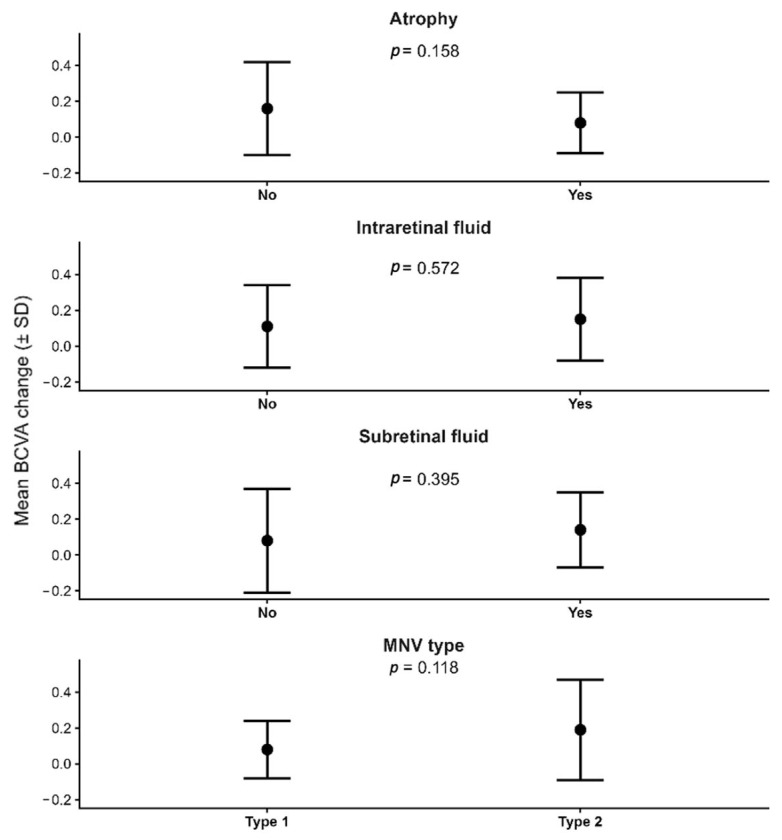
Mean change in best-corrected visual acuity (BCVA) over 12 months according to baseline retinal characteristics (atrophy, intraretinal fluid, subretinal fluid) and macular neovascularization (MNV) type. Points represent mean BCVA change and error bars indicate standard deviation. *p*-values correspond to between-group comparisons for each baseline variable.

Other baseline morphological characteristics—including atrophy, intraretinal fluid, subretinal fluid, and MNV subtype—did not significantly influence BCVA evolution (all *p* > 0.11), although type 2 MNV exhibited a numerically greater but non-significant improvement compared with type 1 (Figure 2).

A multivariable linear regression model including age, sex, baseline BCVA, MNV subtype, presence of SRF, baseline atrophy, and total number of injections identified baseline absence of PVD as the only independent predictor of greater BCVA gain at 12 months (β = −0.27; 95% CI −0.42 to −0.12; *p* = 0.0008). None of the remaining covariates—including MNV type, SRF, baseline atrophy, or treatment burden—reached statistical significance, indicating that vitreous status contributed unique explanatory value beyond conventional anatomical or clinical factors. In clinical terms, this effect corresponds to an adjusted mean benefit of approximately 0.27 logMAR—equivalent to about 13–14 Early Treatment Diabetic Retinopathy Study (ETDRS) letters—meaning that eyes without PVD achieved roughly one and a half additional lines of visual improvement compared with eyes in which PVD was already present. The final model explained 28% of the variance in BCVA change (adjusted R^2^ = 0.28).

### 3.6. Treatment

All patients received intravitreal aflibercept following a treat-and-extend regimen. Treatment intensity varied according to baseline vitreous configuration. Eyes with complete PVD required the highest number of injections over the 12-month period, averaging seven injections, whereas those without PVD or with partial PVD required an average of six injections. This difference was statistically significant (*p* = 0.046), suggesting that eyes with complete vitreous detachment may have a higher treatment demand to maintain disease control.

## 4. Discussion

The key finding of this study is that baseline vitreous status, assessed by ocular ultrasonography, independently predicts functional response to anti-VEGF therapy in neovascular AMD. Eyes without posterior vitreous detachment achieved greater visual improvement over 12 months, while eyes with complete PVD required a higher treatment burden. These associations persisted after adjustment for baseline visual acuity, macular morphology, MNV subtype, and injection number, indicating that vitreous configuration provides prognostic information beyond conventional OCT-derived biomarkers.

### 4.1. Comparison with the Control Group

The comparative analysis between nAMD and control eyes without macular abnormalities provides insight into the potential role of the vitreous disease pathophysiology. Demographic and clinical characteristics were homogeneous between groups, minimizing confounding by age, sex, hypertension, cataract surgery, or AXL, in line with previous studies that emphasize the need to control for these variables when investigating vitreous status in retinal disease [14,15].

A markedly higher proportion of nAMD eyes exhibited absence of PVD (persistent vitreous attachment) compared with controls. This supports the concept of altered physiological detachment mechanisms in nAMD and is consistent with series reporting a high prevalence of anomalous VMA in nAMD [6,7], particularly in “typical” nAMD compared with polypoidal choroidal vasculopathy [16]. Persistent vitreous attachment may be associated with tractional and inflammatory microenvironmental changes; however, mechanistic links (e.g., cytokine gradients or VEGF dynamics) remain speculative and should be considered hypothesis-generating [17,18]. Paired-eye and OCT studies further support colocalization of VMA with the site of MNV [6]. These pathophysiological interpretations should be regarded as hypothesis-generating, as the present study was not designed to directly assess the molecular or biomechanical mechanisms linking vitreous adhesion to neovascular development.

Our finding of fewer partial PVDs in nAMD than in controls contrasts with Krebs et al. [6] and likely reflects differences in definitions and imaging protocols. Methodological heterogeneity—especially in the distinction between VMA, VMT, and partial versus complete PVD—has been highlighted in systematic reviews and meta-analyses and limits comparability across series [9]. While OCT is indispensable for macular disease assessment, its restricted scanning area limits its ability to characterize global vitreous configuration, which may explain part of the heterogeneity observed across previous studies relying exclusively on OCT-based definitions of PVD. This underscores the need for standardized criteria for intermediate stages of vitreous detachment.

Complete PVD was similarly prevalent in both groups, consistent with large population-based cohorts suggesting that complete PVD is primarily an age-related phenomenon rather than a specific marker of nAMD [14,19,20,21,22,23]. However, beyond the simple presence or absence of PVD, the pattern and timing of vitreoretinal interface changes may be relevant. Persistent VMA/VMT has been associated with greater injection burden and poorer early anatomical response in *pro re nata* (PRN) studies, although visual outcomes at 12 months are often similar [24,25]. Under T&E regimens, eyes with VMA tend to require more injections than those with PVD while achieving comparable BCVA and central thickness [26]. In our aflibercept-treated T&E cohort, by contrast, eyes without PVD showed the greatest functional gain, whereas those with complete PVD required more injections, emphasizing that vitreous status should be interpreted as a dynamic biomarker whose impact may vary across treatment protocols and anti-VEGF agents [6,9,25,26,27,28,29].

Overall, these data reinforce that vitreous evaluation in nAMD should go beyond a binary PVD/no-PVD distinction and consider the quality, location, and progression of vitreoretinal adhesions, as well as their potential mechanobiological impact on neovascular development and treatment response [9].

### 4.2. nAMD: Descriptive Clinical Profile

The demographic and clinical profile of our nAMD cohort is broadly consistent with previous epidemiological and clinical reports. As expected, patients were predominantly older adults, reflecting the strong association between aging and nAMD [30,31], and showed a high prevalence of cardiovascular comorbidities, in line with other series of chronic retinal disease [32]. The female predominance observed in our cohort also aligns with population-based studies suggesting a higher burden of nAMD among women [1,33,34].

From a disease-severity perspective, baseline functional and structural features were indicative of active neovascular disease, comparable to populations enrolled in major anti-VEGF clinical trials [2,3,4,5]. The predominance of type 1 macular neovascularization and frequent exudative findings are characteristic of treatment-naïve nAMD [35,36], while the coexistence of baseline atrophy highlights the overlapping degenerative and exudative components commonly observed in advanced disease stages [37]. Prior cataract surgery was common, as expected in an elderly population, and the controversial relationship between phacoemulsification and nAMD progression remains unresolved [38,39,40].

### 4.3. Vitreous Detachment and Clinical Profile in Patients with nAMD

Within the nAMD cohort, age, AXL, systemic comorbidities, and baseline BCVA did not differ significantly across PVD categories. This contrasts with general population data, where complete PVD increases with age [41,42], and supports the notion that nAMD may alter usual PVD dynamics [6,43,44]. Although we observed a numerically higher proportion of women with complete PVD, and hormonal influences on vitreous matrix composition have been postulated [33,34,45], our sample size does not allow firm conclusions regarding sex-related differences.

Structural features such as foveal thickness, intraretinal cysts, SRF, and atrophy showed no statistically significant differences between PVD groups, although partial PVD tended to be associated with thicker maculae, compatible with persistent traction. More importantly, vitreous status was significantly associated with MNV subtype: PVD presence was linked to a higher prevalence of type 1 MNV, whereas type 2 MNV was relatively more frequent in eyes without PVD. OCT-based refinement of VMA within the partial-PVD group further suggested that persistent VMA may favor type 2 neovascularization, consistent with hypotheses suggesting tractional forces and altered cytokine gradients, including VEGF, in neovascular patterning [6,7,40,46,47].

Taken together, these findings support a modulatory role of the vitreoretinal interface on MNV phenotype and highlight the need to incorporate detailed vitreous assessment into the characterization of nAMD.

### 4.4. Vitreous Dynamics and Changes in Clinical Parameters During Treatment

Longitudinal follow-up showed a significant progression toward complete PVD over 12 months, in agreement with hypotheses that PVD evolution in nAMD is an active, disease-modulated process rather than a purely age-driven event [10,43,46,48]. Repeated intravitreal injections, resolution of edema, and vitreous remodeling may all contribute to weakening vitreoretinal adhesion and facilitating detachment.

Anatomically, SRF and IRF decreased markedly during treatment, while macular atrophy increased, consistent with previous reports linking long-term anti-VEGF therapy and chronic disease evolution to atrophic changes [3,47,49]. However, baseline PVD status did not significantly modify atrophy progression, IRF evolution, or the magnitude of foveal thickness reduction, suggesting that these structural trajectories are driven primarily by neovascular control, choroidal perfusion, and retinal pigment epithelium integrity rather than vitreous configuration [3,7,47,50,51,52,53]. Partial and complete PVD were associated with somewhat greater SRF resolution than no PVD, which is compatible with reduced traction and improved fluid clearance [9,10,25,54], but these differences were modest and not consistently reflected in functional outcomes.

### 4.5. Evolution of Visual Acuity

BCVA improved significantly over 12 months in the overall cohort, but the magnitude of gain differed according to baseline vitreous status. Eyes without PVD achieved the greatest visual improvement, followed by those with partial and complete PVD. After OCT-based reclassification, eyes with OCT-defined VMA showed larger functional gains than those without VMA, suggesting a complex relationship between vitreous attachment, neovascular phenotype, and visual response [6,10,46,51,55,56,57]. Importantly, these findings suggest differences in the magnitude and timing of functional improvement rather than in a distinct final anatomical endpoint.

Crucially, our multivariable linear regression model confirmed that absence of PVD at baseline was an independent predictor of greater BCVA gain at 12 months, even after adjusting for age, sex, baseline BCVA, MNV subtype, baseline SRF and atrophy, and injection number. This indicates that vitreous status exerts a functional influence beyond that expected from baseline anatomy or neovascular phenotype alone. The lack of correlation between BCVA gain and foveal thickness reduction, in line with previous work, reinforces the notion that structural normalization does not fully capture functional recovery, which depends on photoreceptor integrity, choroidal perfusion, and retinal remodeling [4,39,47,53].

Other baseline variables, including atrophy, IRF, SRF, and MNV subtype, showed only non-significant trends with respect to functional outcomes. The fact that eyes without PVD—where type 2 MNV was more frequent and visual gains tended to be larger—performed best despite not achieving the greatest SRF resolution illustrates the complexity of the interplay between vitreous status, MNV phenotype, fluid patterns, and visual function [6,7].

### 4.6. Treatment Burden According to Vitreous Status

All patients were treated with aflibercept in a T&E regimen, and baseline vitreous status influenced injection burden. Eyes with complete PVD required significantly more injections than those without PVD or with partial PVD, suggesting reduced treatment efficiency or greater residual angiogenic drive in this subgroup. Prior studies have reported both increased treatment burden in eyes with VMA/VMT and variable results for PVD, depending on the anti-VEGF agent and regimen [6,24,26,28,29,58].

Our findings add nuance to this literature by showing that vitreous status modulates both functional trajectory and treatment burden: absence of PVD was associated with greater visual gain, whereas complete PVD was associated with higher injection requirements. Differences in how PVD and VMA are defined (OCT vs. ultrasonography), the distribution of MNV subtypes, and protocol-specific extension criteria likely contribute to discrepant results across studies. Nonetheless, the present data support the concept that vitreous status is a relevant determinant of both functional response and treatment burden and should be considered within a broader, individualized therapeutic strategy for nAMD.

If validated in larger cohorts, ultrasonography-based vitreous assessment could contribute to risk stratification frameworks and inform future studies on individualized dosing strategies; at present, it should be viewed as an adjunct imaging biomarker rather than a tool for treatment planning. These findings should be interpreted within the context of an aflibercept treat-and-extend regimen and require validation under other anti-VEGF agents and dosing strategies.

Although OCT provides high-resolution information on the vitreomacular interface, ocular ultrasonography is particularly well suited to evaluate global posterior vitreous detachment and vitreous mobility, which cannot be reliably assessed with macular OCT alone. In this context, ultrasonography is justified in selected prognostic and research scenarios, such as vitreous phenotyping, prognostic stratification of functional trajectory and treatment burden under standardized anti-VEGF regimens, evaluation of clinical–anatomical discordance (good OCT anatomy with suboptimal functional improvement), and dynamic assessment of intermediate PVD stages in which macular OCT may underestimate the overall vitreous configuration.

Accordingly, ultrasonography should be regarded as a complementary prognostic imaging biomarker, providing contextual information rather than directly guiding routine treatment decisions, and particularly valuable in studies investigating modifiers of treatment response and vitreoretinal biology in neovascular AMD.

### 4.7. Limitations

This study has several limitations. First, although the prospective design ensured standardized imaging and follow-up, subgroup sample sizes were modest, particularly when stratifying partial and complete posterior vitreous detachment, which may limit the detection of subtle differences. Second, ultrasonography and OCT assess complementary but non-identical aspects of the vitreoretinal interface; therefore, misclassification of intermediate PVD stages, although minimized by high interobserver agreement, cannot be fully excluded. In particular, dynamic B-scan ultrasonography is well validated for global PVD assessment and posterior hyaloid mobility but has limited spatial resolution for defining focal vitreomacular adhesion, for which OCT remains the reference technique. In routine clinical practice, a macular OCT–based assessment of the vitreomacular interface is often sufficient, accepting a known limitation in the evaluation of global PVD status in exchange for greater efficiency and reduced examination time. Third, the OCT-based refinement of partial PVD categories represents a necessary but inherently post hoc segmentation. Fourth, all patients were treated with aflibercept under a treat-and-extend regimen; consequently, the findings may not be directly generalizable to other anti-VEGF agents or treatment protocols. Finally, the 12-month follow-up provides valuable information on early and mid-term outcomes but does not capture long-term atrophic progression. Larger multicenter cohorts with standardized definitions of vitreous stages will be important to validate these findings.

## 5. Conclusions

This study demonstrates that the vitreomacular interface is a meaningful structural modifier in neovascular AMD. Compared with age-matched controls, nAMD eyes showed a markedly higher prevalence of absent posterior vitreous detachment, suggesting altered vitreous separation dynamics in the neovascular phenotype. Within the nAMD cohort, vitreous status influenced neovascular patterning: the presence of PVD was associated with a higher frequency of type 1 macular neovascularization, whereas type 2 lesions were more common in eyes without PVD. Baseline absence of PVD (persistent vitreous attachment) modulated therapeutic outcomes, with eyes without PVD achieving the greatest visual gain, while eyes with complete PVD required a higher number of aflibercept injections under a treat-and-extend regimen. Although final anatomical outcomes were broadly similar across groups, these findings indicate that vitreous configuration primarily influences the functional trajectory of visual recovery and treatment burden, rather than the ultimate anatomical endpoint.

In this context, incorporating vitreous assessment by integrating OCT-based macular evaluation with ultrasonography-defined global vitreous status may enhance prognostic stratification of functional response and treatment intensity in neovascular AMD. At present, ocular ultrasonography should be regarded as a complementary imaging biomarker, particularly valuable in research settings and in selected clinical scenarios where a more complete characterization of vitreous configuration may provide additional prognostic context. While not intended to directly guide routine treatment decisions, this approach may help explain inter-individual variability in functional response and treatment requirements under standardized anti-VEGF therapy and warrants validation in larger, standardized cohorts.

## Figures and Tables

**Table 1:** Demographic and clinical comparison between patients with nAMD (N = 51) and controls (N = 65).

Characteristics	nAMD (N = 51)	Controls (N = 65)	*p*-Value
**Age (years)**	79 ± 9	79 ± 4	0.790
**AXL (mm)**	23.25 ± 1.31	23.29 ± 0.68	0.860
**Female—n (%)**	31 (60.8)	34 (52.3)	0.361
**HTN—n (%)**	30 (58.8)	33 (52.4)	0.492
**Previous cataract surgery—n (%)**	27 (52.9)	29 (46.0)	0.463

Abbreviations: AXL: axial length; HTN: arterial hypertension; nAMD: neovascular age-related macular degeneration.

**Table 2:** Descriptive analysis of clinical and demographic characteristics in patients with nAMD (N = 51).

Characteristics	(N = 51)
**Age (years)**	79 ± 9
**AXL (mm)**	23.25 ± 1.31
**BCVA (Snellen)**	0.27 ± 0.22
**Foveal thickness (µm)**	409 ± 122
**Female—n (%)**	31 (60.8)
**Right eye—n (%) **	23 (45.1)
**HTN—n (%)**	30 (58.8)
**Diabetes—n (%)**	9 (17.6)
**Type 1 macular neovascularization—n (%)**	28 (54.9)
**Subretinal fluid—n (%)**	40 (78.4)
**Baseline atrophy—n (%)**	20 (39.2)
**Intraretinal cysts—n (%)**	21 (41.2)
**Previous cataract surgery—n (%)**	27 (52.9)

Abbreviations: AXL: axial length; BCVA: best-corrected visual acuity; HTN: arterial hypertension.

**Table 3:** Clinical and demographic comparison of patients with nAMD according to PVD status.

Characteristics	No PVD (n = 13)	Partial PVD (n = 12)	Complete PVD (n = 26)	*p*-Value
**Age (years)**	79 ± 6	76 ± 15	81 ± 7	0.871
**AXL (mm)**	22.87 ± 0.95	23.57 ± 1.65	23.22 ± 1.23	0.280
**BCVA (Snellen)**	0.21 ± 0.12	0.35 ± 0.23	0.27 ± 0.25	0.274
**Foveal thickness (µm)**	387 ± 108	431 ± 134	409 ± 125	0.673
**Female—n (%)**	7 (53.8)	5 (41.7)	19 (73.1)	0.153
**Right eye—n (%)**	6 (46.2)	4 (33.3)	13 (50.0)	0.628
**HTN—n (%)**	9 (69.2)	4 (33.3)	17 (65.4)	0.119
**Diabetes—n (%)**	3 (23.1)	2 (16.7)	4 (15.4)	0.834
**Type 1 MNV—n (%)**	4 (30.8)	7 (58.3)	17 (65.4)	0.118
**Intraretinal cysts—n (%)**	8 (61.5)	3 (25.0)	10 (38.5)	0.165
**Subretinal fluid—n (%)**	11 (84.6)	10 (83.3)	19 (73.1)	0.636
**Baseline atrophy—n (%)**	5 (38.5)	2 (16.7)	13 (50.0)	0.147
**Previous cataract surgery—n (%)**	8 (61.5)	4 (33.3)	15 (57.7)	0.290

Abbreviations: AXL: axial length; BCVA: best-corrected visual acuity; HTN: arterial hypertension; MNV: macular neovascularization; PVD: posterior vitreous detachment.

**Table 4:** Demographic and clinical characteristics of patients with nAMD grouped by presence or absence of PVD.

Characteristics	No PVD (n = 13)	PVD (n = 38)	*p*-Value
**Age (years)**	79 ± 6	79 ± 10	0.731
**AXL (mm)**	22.87 ± 0.95	23.36 ± 1.39	0.158
**BCVA (Snellen)**	0.23 ± 0.13	0.30 ± 0.32	0.252
**Foveal thickness (µm)**	387 ± 108	431 ± 134	0.473
**Female—n (%)**	7 (53.8)	24 (63.2)	0.553
**Right eye—n (%)**	6 (46.2)	21 (55.3)	0.929
**HTN—n (%)**	9 (69.2)	21 (55.3)	0.377
**Diabetes—n (%)**	3 (23.1)	6 (15.8)	0.552
**Type 1 MNV—n (%)**	4 (30.8)	24 (63.2)	0.043
**Intraretinal cysts—n (%)**	8 (61.5)	13 (34.2)	0.084
**Subretinal fluid—n (%)**	11 (84.6)	29 (76.3)	0.530
**Baseline atrophy—n (%)**	5 (38.5)	23 (60.5)	0.207
**Previous cataract surgery—n (%)**	8 (61.5)	19 (50.0)	0.472

Abbreviations: AXL: axial length; BCVA: best-corrected visual acuity; HTN: arterial hypertension; MNV: macular neovascularization; PVD: posterior vitreous detachment.

## Data Availability

The data presented in this study are available on request from the corresponding author.

## Abbreviations

The following abbreviations are used in this manuscript:

AMD	Age-related Macular Degeneration
AXL	Axial Length
BCVA	Best-Corrected Visual Acuity
ETDRS	Early Treatment Diabetic Retinopathy Study
HTN	Hypertension
IRF	Intraretinal Fluid
MNV	Macular Neovascularization
OCT	Optical Coherence Tomography
PRN	Pro Re Nata
PVD	Posterior Vitreous Detachment
SRF	Subretinal Fluid
T&E	Treat-and-Extend
VEGF	Vascular Endothelial Growth Factor
VMA	Vitreomacular Adhesion
VMT	Vitreomacular Traction
